# An Open Source “Smart Lamp” for the Optimization of Plant Systems and Thermal Comfort of Offices

**DOI:** 10.3390/s16030338

**Published:** 2016-03-07

**Authors:** Francesco Salamone, Lorenzo Belussi, Ludovico Danza, Matteo Ghellere, Italo Meroni

**Affiliations:** Construction Technologies Institute, National Research Council of Italy (ITC-CNR), Via Lombardia, 49, 20098 San Giuliano Milanese (MI), Italy; belussi@itc.cnr.it (L.B.); danza@itc.cnr.it (L.D.); ghellere@itc.cnr.it (M.G.); meroni@itc.cnr.it (I.M.)

**Keywords:** open source, Arduino, App Inventor, DIY, IoT, control system, environmental monitoring system, building automation, thermal comfort, energy saving

## Abstract

The article describes the design phase, development and practical application of a smart object integrated in a desk lamp and called “Smart Lamp”, useful to optimize the indoor thermal comfort and energy savings that are two important workplace issues where the comfort of the workers and the consumption of the building strongly affect the economic balance of a company. The Smart Lamp was built using a microcontroller, an integrated temperature and relative humidity sensor, some other modules and a 3D printer. This smart device is similar to the desk lamps that are usually found in offices but it allows one to adjust the indoor thermal comfort, by interacting directly with the air conditioner. After the construction phase, the Smart Lamp was installed in an office normally occupied by four workers to evaluate the indoor thermal comfort and the cooling consumption in summer. The results showed how the application of the Smart Lamp effectively reduced the energy consumption, optimizing the thermal comfort. The use of DIY approach combined with read-write functionality of websites, blog and social platforms, also allowed to customize, improve, share, reproduce and interconnect technologies so that anybody could use them in any occupied environment.

## 1. Introduction

Over the past years several shared projects and low-cost alternative technologies have appeared and developed, bringing end users closer to electronics in a simple and fast way [1,2,3]. In particular, through the Do-It-Yourself (DIY) approach, the user becomes also the maker of these technologies, removing structural, technological and economical obstacles [4]. The spread of this movement has led to the proliferation of devices always connected to a communicating-actuating network, *i.e.*, a web of objects connected to the network and interconnected to each other, named Internet of Things (IoT) [5] starting a technological revolution. After the agricultural and the industrial revolutions, the information age, the so called Third Wave [6], draws upon the read/write functionality of the Internet and digitally-driven design/manufacture, to enable ordinary people to invent, design, make and, sometimes, sell goods and services [7]. Anybody at any location could carry out the principles of the DIY philosophy [8,9,10] through enabling technologies, for example Arduino [11] or Genuino [12].

Today, websites like OpenMaterials [13], Instructables [14], Make Magazine [15], Adafruit [16], Sparkfun [17], Cubify [18], Thingiverse [19] allow the quick sharing of smart, low cost and DIY approach-based solutions among makers or enthusiasts. The several licenses provided by Creative Commons [20] allow protection of the knowledge and creativity of the projects’ authors. In this paper the DIY approach has been applied to a smart system integrated into a desk lamp similar to those used in offices. This device, called Smart Lamp, allows the indoor thermal comfort to be controlled and optimized, through direct interaction with the Heating, Ventilation, and Air Conditioning (HVAC) system installed in the room.

Thermal comfort in indoor environments has been a research field for decades [21]. The problem of thermal comfort in the workplace is still a hot topic [22,23,24,25]. Studies indicate how thermal comfort can improve occupants’ satisfaction and productivity and simultaneously decrease the energy consumption of buildings [25]. The different facets of the problem have been analysed and some solutions have been proposed over the years. Recently, some studies show how the IoT approach has been applied to the physical environment aimed at improving the user’s satisfaction [26] and innovative projects were born in order to create smart environment based on open source and hardware devices [27]. The open source approach has an enormous impact on current technology and has changed the way in which many tech companies do business and therefore our society [28,29,30]. Following this path, the new smart device presented below was implemented in order to prevent low occupant satisfaction and high energy costs, demonstrating how these new technologies can be easily integrated in the real built environment and improve the well-being of end users. The system has been realized using for the most part open source software [31,32,33,34]. The Smart Lamp was built using an Arduino Mega board, a DHT22 sensor to detect the environmental variables (temperature, relative humidity), a Real Time Clock (RTC) module, an Infrared (IR) LED to connect to the HVAC system and a Bluetooth module to transfer data. An appropriately designed case was realized using a 3D printer. As far as the microcontroller is concerned, several manufacturers such as Parallax Inc., Coridium Corporation, FTDI, Picaxe, Arduino, as well as many others, have proposed quite popular and inexpensive solutions. Among them, Arduino boards offer one critical advantage: the open source philosophy (both hardware and software), which capitalizes on the massive non-expert community which is growing around the Arduino concept. The analyses to evaluate the performance of the DHT22 sensor [35] highlighted the good reliability of this sensor compared to much more expensive professional sensors, with a complete data sheet and a calibration report. The RTC module was necessary for the correct execution of the algorithms and also to implement the data logging functionality. The IR-LED connects the Smart Lamp to the HVAC system and the Bluetooth module to a smartphone or a tablet. The case was built based on the characteristics of a real lamp. Some components were designed and manufactured with a PowerWasp 3D printer. This 3D printer implements the Fused Deposition Modeling (FDM) technology and uses polylactide (PLA) for printing. PLA is one of the most eco-friendly 3D available printing materials made from renewable resources (corn-starch) and requires less energy for processing with respect to traditional petroleum-based plastics.

Even the developed hardware system is available online as an open source project. It is a way to make accessible and check how this device allows to optimize the thermal comfort and the energy consumption, evolving over time relying on end-users’ participation.

## 2. Smart Lamp, Hardware and Software

### 2.1. Architecture

The Smart Lamp looks like an ordinary halogen desk lamp [36] that implements an active function of adjustment of indoor thermal comfort and optimization of energy consumption by interfacing with an air conditioning system. This object was chosen as the basis of integration of a smart control system because it can be found in most working environments, particularly in offices. This device can be easily replicated thanks to the instructions and freely available information [37,38].

**Figure 1:**
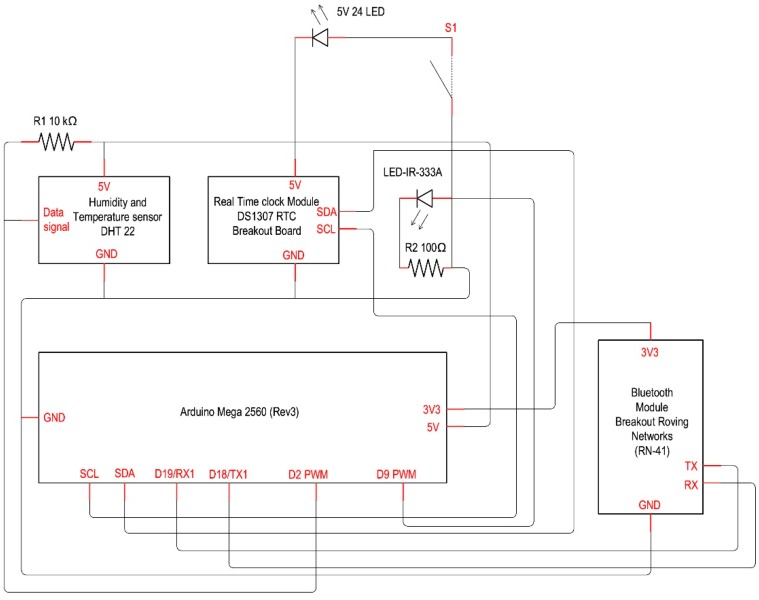
Smart Lamp: wiring diagram.

The hardware (Figure 1) consists of the following components:

- Arduino MEGA 2560 r3 with “sandwich connected” wireless shield [39,40];
- DHT22, temperature and relative humidity sensor [35];
- RTC module based on DS1307 chip [41];
- LED IR [42];
- 24 LEDs panel board [43];
- Bluetooth module (optional) [44].

The choice of the Arduino MEGA PCB based on the ATmega2560 microcontroller is due to the possibilities offered by this device for managing the large amount of data necessary to send infrared signals to the HVAC unit. The board has 54 input/outputs (I/O) (14 of which can be used as Pulse-Width Modulation (PWM) outputs), 16 analog inputs, four Universal Asynchronous Receiver-Transmitter (UART), 16 MHz quartz, USB connection, power jack, programming header for In-Circuit Serial Programming (ICSP) and reset button.

The Wireless SD shield allows one to save the data into the Micro SD card placed in the on-board slot. It allows an Arduino board to communicate wirelessly using a wireless module too. It’s considered this shield instead of Wi-Fi ones because one future upgrade regards the possibility to connect this device to a coordinator through radio frequency module (XBee S2 type) in a Wireless Sensor Network (WSN) with some others device. The open source approach of this project gives to everyone the possibility to consider a Wi-Fi shield in substitution of this one.

The DHT22 sensor is small in size and operates with a supply voltage between 3.3 and 6 V. It communicates both data (temperature and relative humidity) through a single pin. It is able to measure temperatures with a range between −40 and 80 °C, with an accuracy of less than ±0.5 °C, and to detect the relative humidity between 0% and 100%, with an accuracy of ±2%. The sensor provides fully digital calibrated outputs for the two measurements. The protocol must be implemented in the firmware of the microcontroller and it is not compatible with the 1-Wire^®^ protocol.

The RTC module was developed around the DS1307 Integrated Circuit (IC) by Maxim. The DS1307 IC is a Binary-Coded Decimal (BCD) low consumption clock/calendar with 56 bytes of RAM powered by a back-up battery. Addresses and data are transmitted serially via the bidirectional I2C bus. The clock/calendar provides information on seconds, minutes, hours, day, month and year. The end of the month is automatically adjusted for months with less than 31 d, including the correction for leap years. The clock can work in 24 and 12 h format with AM/PM indication. The DS1307 has a power-sense integrated circuit that detects the power failure and automatically enables the power supply through the backup battery.

The LED is IR-type, with a diameter of 5 mm and it is characterized by a peak wavelength, λp, equal to 940 nm.

The BlueSMiRF Gold Bluetooth module allows one to easily transmit the signal over distances of less than 100 meters. It has a low power consumption of less than 25 mA with operating voltage between 3.3 and 6 V. The operating frequency is 2.402 ÷ 2.480 GHz. It uses the RN-41 chip for the connection. It has a default BAUD rate of 115,200.

The electronic architecture has been integrated in the lamp base. This choice prevents the lamp from tipping over and allows to exploit the interior space for the housing of the components. The lighting element is a “cold light” LED board in place of the original halogen bulb, in order to minimize the energy consumption of the lamp.

**Figure 2:**
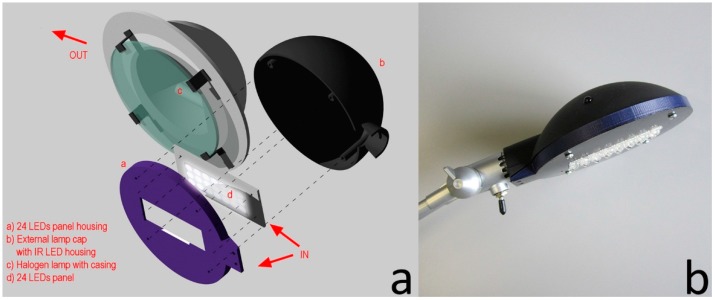
Smart Lamp: (**a**) assembly diagram of the lamp housing; (**b**) as installed.

The construction of the Smart Lamp has required a series of adjustments and the manufacturing of two distinct cases: the housing for the installation of the LED panel and the infrared emitter and a new base in which the elements described above are integrated. Both cases are designed in 3D CAD format and printed with a 3D printer with PLA. The new lamp housing (Figure 2) has the same shape as the original one and provides the fixing of the LED panel with screws on the back.

**Figure 3:**
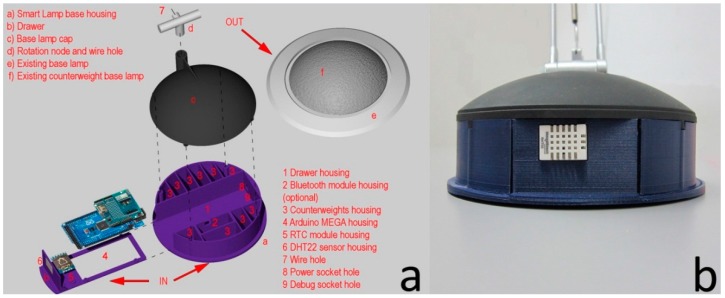
Smart Lamp: (**a**) assembly diagram of the base; (**b**) as installed.

The new base (Figure 3) consists of a flat cylinder with an internal subdivision in blocks. The biggest central block is equipped with a removable drawer allowing the maintenance of the electronic devices.

The electronic components (Arduino MEGA Arduino Wireless Shield, RTC module and sensor DHT22) are placed inside this base (Figure 3), except for the optional Bluetooth module which is placed in a separate compartment (No. 2 in Figure 3). Arduino MEGA, Arduino Shield (“sandwich” connected) and RTC are fixed to the drawer. The sensitive component of the DHT22 sensor is outward-facing to detect the environmental variables. Furthermore, a series of small compartments intended to contain the counterweights for balancing the lamp are provided.

**Figure 4:**
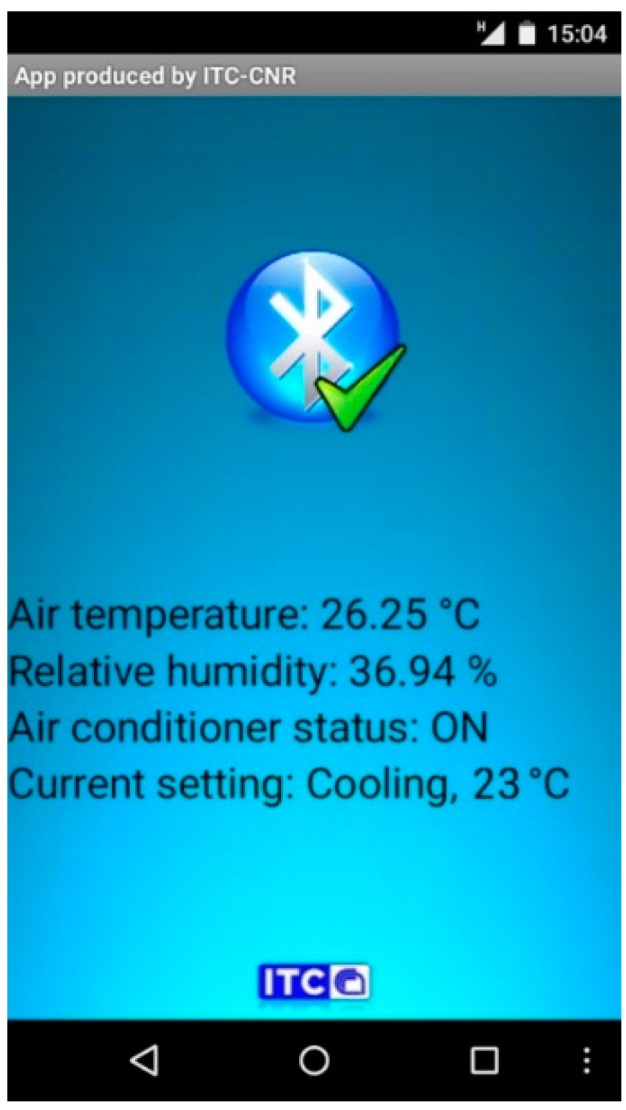
Application for mobile devices (Android OS).

An app for Android devices was created with the aid of MIT App Inventor, a visual programming blocks language for Android OS [45]. The wireless shield mounted on the Arduino MEGA allows to record the data on a micro-SD card. The Bluetooth module allows to send the data to an app (Figure 4) to visualize the temperature and the relative humidity data and to verify the operating status of the HVAC system. It is used a Serial Port Profile (SPP): the Bluetooth module work as a serial transmission connector. All serial printed data are streamed to the smartphone. All data can be stored in a cloud server using the Wi-Fi or the data connection of the smartphones.

Although there are other applications that allow you to view and interact with smart devices, the use of a “drag-and-drop” visual programme language like app inventor allows to an inexperienced novice to easily create this app and modify to suit specific needs.

### 2.2. Management Algorithm of the HVAC System

**Figure 5:**
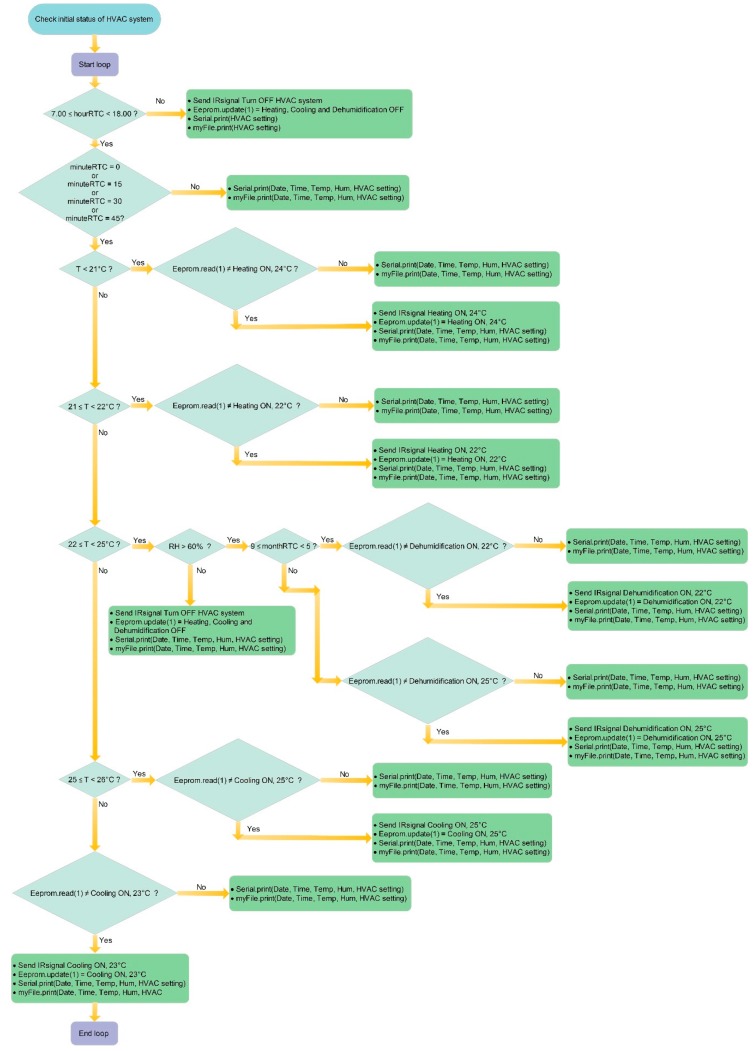
Smart Lamp: management algorithm of HVAC system.

The code of Arduino MEGA board implements the logic control shown in Figure 5. The HVAC system is connected to an electrical device which automatically switches off the thermal plant from 7.00 pm to 7.00 am, from Friday to Monday. Users can then use the HVAC system during the working hours changing the operating settings manually. The Smart Lamp allows one to manage the system from 7.00 am to 6.00 pm for the best thermal comfort conditions, recording the indoor temperature, the relative humidity and the setup of the HVAC system every minute on a microSD memory. Every 15 min, the control system checks the indoor environmental values and performs an actuation in terms of activation/deactivation of the HVAC system, if the setting values are different from what expected. The other controls prevent the Smart Lamp from sending the same command after 15 min if the environmental conditions are not changed, through an Electrically Erasable Programmable Read-Only Memory (EEPROM). So for example, if the ambient temperature is lower than 21 °C, the microcontroller reads the value (Eeprom.read) stored in the cell 1 of the EEPROM. If this value is equal to that corresponding to the “heating ON, 24 °C”, the microcontroller merely write via serial (Serial.print) and on the memory support (myFile.print) the data relating to the date, time, air temperature, relative humidity and HVAC setting; otherwise, the microcontroller also provides to send the infrared appropriate code (Send IRsignal) to the air conditioner (in this case, heating ON, 24 °C) so as to change the setting, and to update the value (Eeprom.update) stored in the cell 1 of the EEPROM.

## 3. Smart Lamp Installation

**Figure 6:**
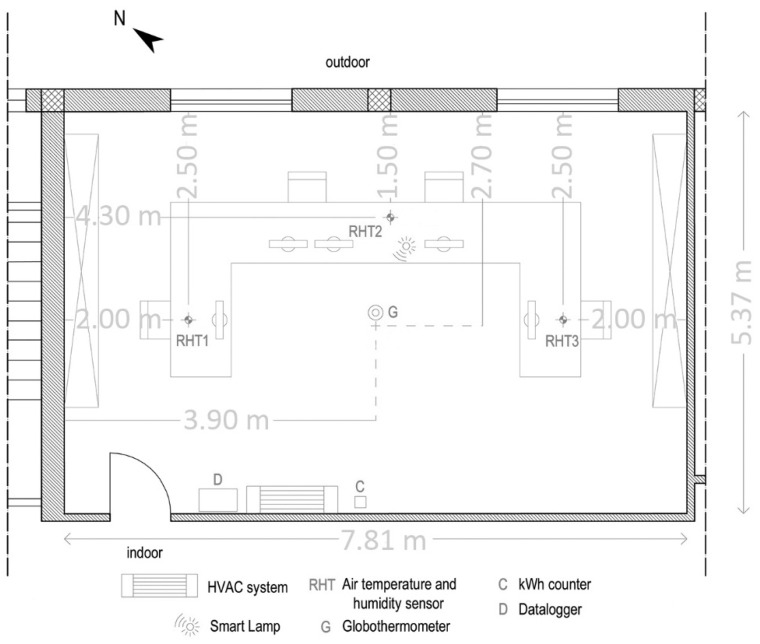
Office plan and sensors distribution.

The Smart Lamp was installed in an office located on the first and top floor of about 42 m^2^ (7.81 m × 5.37 m) normally occupied by four users. The office (Figure 6) is equipped with a HVAC system. Three integrated air temperature and relative humidity sensors (RHT), equally distributed along the 7.81 m long side, a globe thermometer (G) and an energy counter (C) ABB OD 1365 were installed, all connected to a data logger (D): the environmental variables were detected every 10 s and averaged every minute. All data are stored on mass storage device.

**Figure 7:**
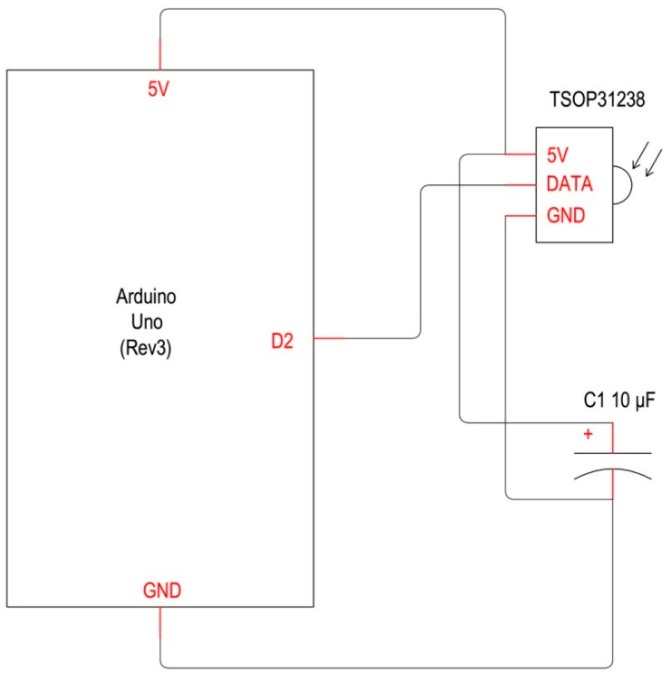
IR signals acquisition system: wiring diagram.

A reverse engineering process allowed to acquire the codes that the remote control sends to the HVAC system, through few hardware elements (Figure 7) and one software:

- Arduino UNO r3 with “sandwich connected” wireless shield [40,46];
- TSOP31238 IR receiver [47];
- AnalysIR software [48].

A TSOP31238 infrared receiver that works in the range of 840 and 960 nm was connected to Arduino UNO r3 PCB. It is sensitive to long-distance transmission and it has a carrier frequency comprised between 30 and 56 KHz. The acquisition frequency of the HVAC system is 38 kHz. The infrared signals picked up by the hardware system described above were recorded and analysed using the software for Windows PCs, AnalysIR [48]. Though this system seven signals, corresponding to particular settings of the HVAC, have been recorded:

- Heating on, 24 °C;
- Heating on, 22 °C;
- Dehumidification on, 22 °C;
- Dehumidification on, 25 °C;
- Cooling on, 23 °C;
- Cooling on, 25 °C;
- A/C system OFF.

These signals were embedded within the code described above.

## 4. Experimentation: Results and Discussion

The levels of indoor thermal comfort and the associated energy consumption for the considered office were assessed. A total of 14 summer working days were analysed, divided into two periods: the former between 30 June and 8 July 2015, with HVAC manual control and the latter between 9 July and 17 July 2015 with automatic control provided by the Smart Lamp.

### 4.1. Thermo-Hygrometric Comfort

The thermo-hygrometric comfort was assessed following the methodology of the ASHRAE 55-2013 [49]. This standard describes a graphical method for the assessment of the indoor thermal comfort by identifying in a psychometric chart two comfort areas for different clothing levels (defined as an index of clothing; typical values are 1 clo, in winter, and 0.5 clo, in summer). The summer comfort zone is considered to be equal to 0.5 clo.

**Figure 8:**
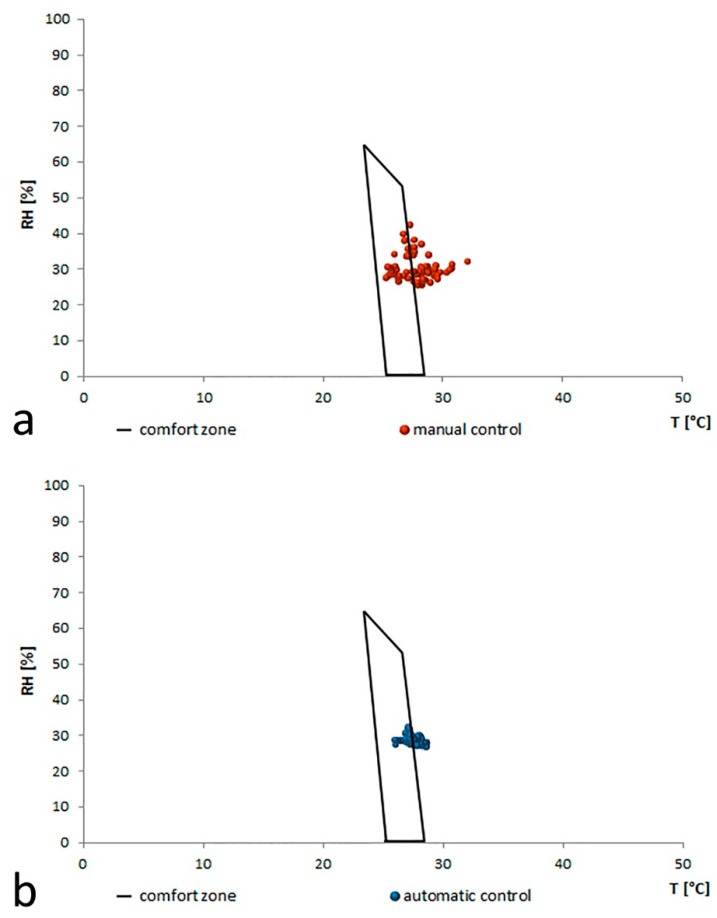
Graphical method of comfort zones: (**a**) manual control; (**b**) automatic control.

Data recorded from 9.00 am to 6.00 pm for the two periods are represented in a graph where the x-axis shows the internal air temperature (T) and the y-axis the relative humidity (RH). The comfort zone delimiting the set of points that ensure a good level of comfort in summer conditions (0.5 clo), is marked as a black line in Figure 8. It can be noted how the red points, referred to the manual control of the HVAC system (Figure 8a), is more dispersed and cover a large area: only 35% of the values falls within the comfort zone. Some points outside the polygon are away from the boundary line with a value even higher than 32 °C. The blue points related to automatic control provided by the Smart Lamp (Figure 8b) are placed and concentrated within the comfort zone—about 65% fall within the comfort zone. It can also be noted that the remaining points outside the polygon are not very far from the boundary line but densely concentrated just along the dividing line with the maximum recorded value of just over 28.5 °C.

### 4.2. Electrical Consumption

**Figure 9:**
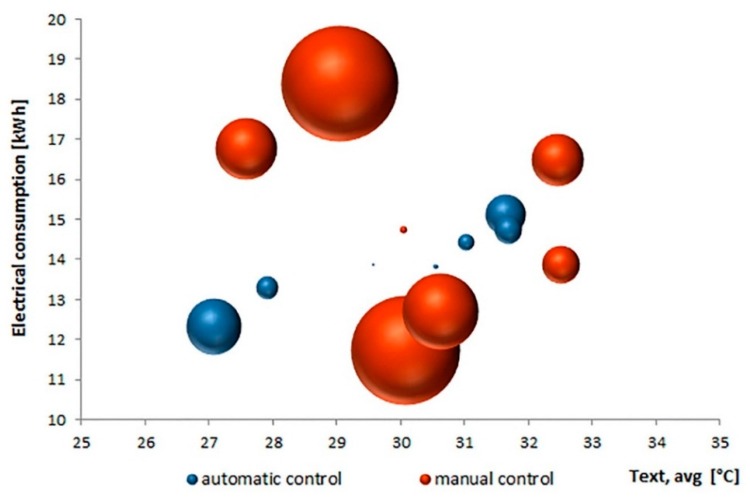
Energy consumption as a function of external average temperature.

The analysis of the data related to the electrical consumption recorded by the energy meter connected to the data logger allows to verify the most efficient control (Figure 9) as a function of the daily average external temperature variation.

The daily average consumption related to the automatic control is equal to 13.96 kWh with an average external temperature equal to 29.91 °C (standard deviation of 0.87 kWh and 1.69 °C, respectively). With manual control, the daily average consumption is equal to 14.97 kWh with an average external temperature equal to 30.30 °C (standard deviation of 2.2 kWh and 1.64 °C, respectively). The area of the spheres of Figure 9 represents the percentage variation of the daily cumulative consumption compared to the mean value of the period. The overall saving provided by the automatic control is slightly higher than 7%. In this case, the linear dependence of the daily cumulated consumption from the average value of external temperature is confirmed by a correlation value (R^2^) equal to 0.91: energy consumption increases with the increasing of the average outdoor air temperature as would be expected in summer conditions [50,51,52]. Instead, in the first case the R^2^ index is equal to 0.1, which is symptomatic of a non-linear dispersion.

## 5. Conclusions

The system, implemented following the DIY philosophy and the use of open hardware and low-cost sensors, allows the indoor thermal comfort to be independently managed. This automatic DIY HVAC control system is an extremely flexible solution. The analysis conducted so far demonstrates how it is possible to optimally manage the indoor thermal comfort and energy consumption because the Smart Lamp, manufactured using a desk lamp, some electronics components and two 3D printed cases, interacts directly with the HVAC system wireless through the IR LED. The hardware and software architecture based on Arduino board and the information for the 3D printer are available for download through website so it is easy to replicate and to customize this solution to specific needs.

The Smart Lamp project is implemented in the field of the Internet of Things for the built environment. The potential of this basic device is confirmed by tests in real working conditions. The characteristics so far described and analysed allow a wide field of application aimed at improving users’ satisfaction and energy consumption of buildings. Current researches are focus on the interaction between the thermal comfort quality and the personal control of users, showing how the micro-climatic conditions play a fundamental role in users’ satisfaction [53,54]. Thanks to the easy reproducibility and application the Smart Lamp could be applied to manage the punctual micro-climatic conditions of a single workplace improving the indoor quality.
